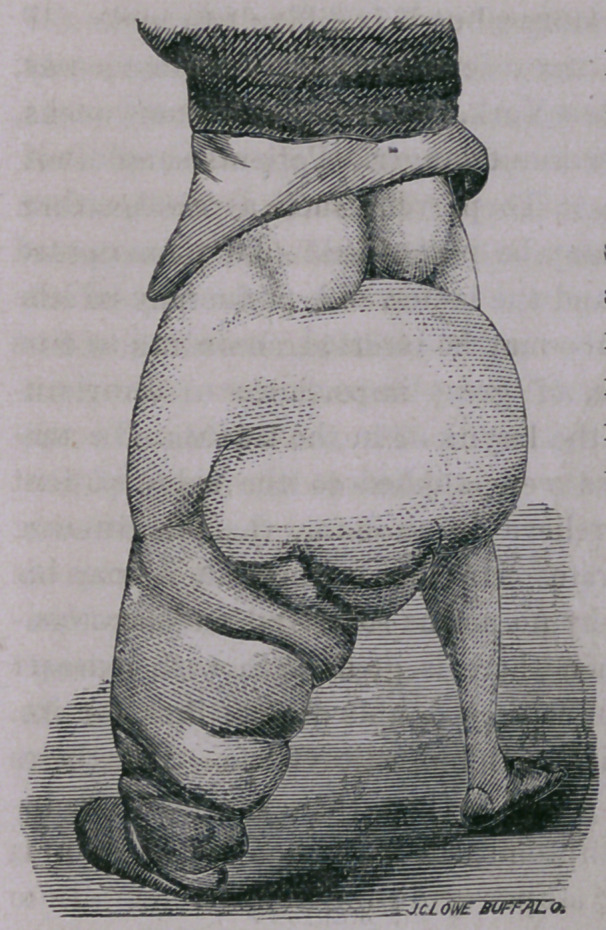# A Case of Elephantiasis Arabum

**Published:** 1871-01

**Authors:** H. D. Ingraham

**Affiliations:** Kennedy, N. Y.


ART. V.—*A Case of Elephantiasis Arabum.* By H. D. INGRAHAM, M. D., of Kennedy, N. Y.

The subject of the following sketch, Mr. A. K. Stockwell, a native of this state, was born April 22d, 1838. His ancestors, both paternal and maternal, were hardy, rugged people; and his brothers and sisters (of which there are a number) have a like healthy and firm constitution. His own health has also always been good. After he became old enough to labor he worked in the lumber woods most of his time, making one or two trips, and occasionally more, down the Alleghany and Ohio Rivers each year. Like all other lumbermen, he worked hard, was in the water considerably, and drank a fair allowance of whisky; yet his health was good all this time, and he had no febrile symptoms.

His leg commenced to enlarge in the sping of 1858, he being then twenty years of age. It has continued to enlarge ever since. In the Fall of 1859 he was, for three or four weeks, under the care of Prof. Frank H. Hamilton, then of Buffalo, who succeeded in the reduction of the enlarged limb to about its normal size. Being uneasy, and tiring of hospital discipline, the patient went away, discontinuing treatment. After this his leg has gradually and constantly enlarged, except for a period ot five weeks, which occurred six years since, when he had typhoid fever, which was such as to confine him to his bed thus long. During the period of this illness, his leg nearly recovered its natural size. Upon convalescing, the leg again commenced enlarging, and soon the under and back side of the enlargement above the knee began to discharge an offensive fluid. This discharge has been constant in occurrence ever since, though of variable amount.

The accompanying cuts have been prepared from photographs, and very faithfully represent the appearance of the case.

The cuticle of the upper part of the thigh is thickened, but is not scaly or of darker color than natural, but from above downwards the skin grows harder, scaly and of darker color, so that the skin of the foot very closely resembles that of the elephant.

The limb now measures, at its principal points of variation, fifty-seven, twenty-nine, thirty-six and thirty inches; the measurement of the instep is twenty-two inches; that of the foot sixteen. The nates are of natural size, as also the right testicle, while the scrotum is fourteen inches in circumference.

The patient is the eldest of a family of seven children. His weight was formerly from one hundred and thirty-five to one hundred and forty pounds, while he now weighs from two hundred and eighty to three hundred pounds.

This is probably as perfect specimen of the disease known as Elephantiasis as is to be found in any country, the nature and causes of which remain but very imperfectly understood.

## Figures and Tables

**Figure f1:**
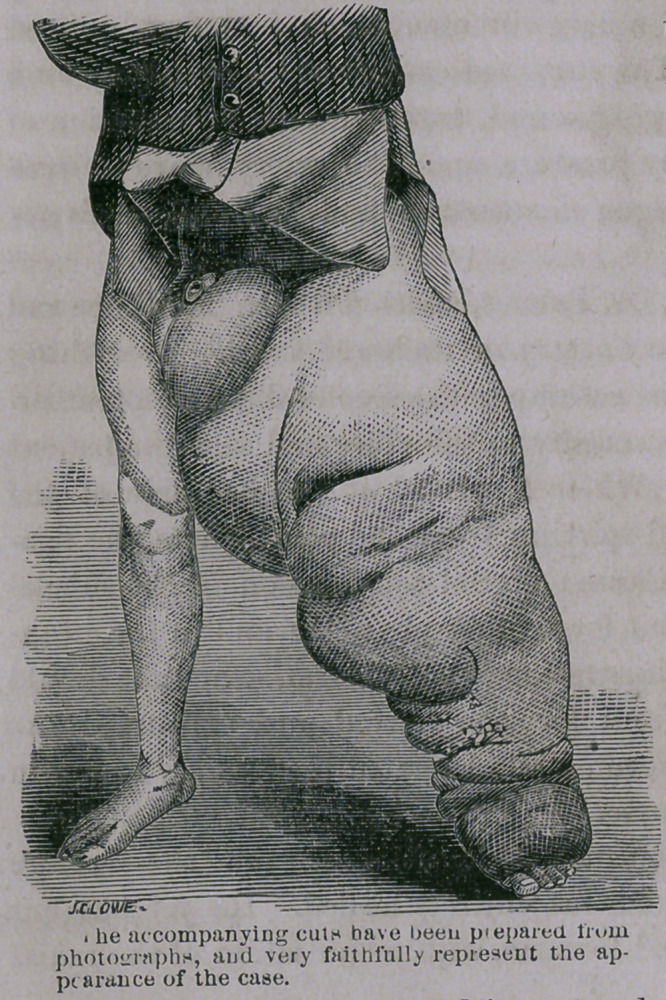


**Figure f2:**